# Development of an In-Pipe Inspection Robot for Large-Diameter Water Pipes

**DOI:** 10.3390/s24113470

**Published:** 2024-05-28

**Authors:** Kwang-Woo Jeon, Eui-Jung Jung, Jong-Ho Bae, Sung-Ho Park, Jung-Jun Kim, Goobong Chung, Hyun-Joon Chung, Hak Yi

**Affiliations:** 1 Korea Institute of Robotics and Technology Convergence, Pohang 37666, Republic of Korea; jeonkw@kiro.re.kr (K.-W.J.); ejjunmg@kiro.re.kr (E.-J.J.); jongho.bae@kiro.re.kr (J.-H.B.); psh84@kiro.re.kr (S.-H.P.); jjkim@kiro.re.kr (J.-J.K.); goobongc@kiro.re.kr (G.C.); 2 Department of Mechanical Engineering, Kyungpook National University, Daegu 41566, Republic of Korea; yihak@knu.ac.kr

**Keywords:** in-pipe robot design, defect detection, autonomous driving, posture control

## Abstract

This paper describes the development of an in-pipe inspection robot system designed for large-diameter water pipes. The robot is equipped with a Magnetic Flux Leakage (MFL) sensor module. The robot system is intended for pipes with diameters ranging from 900 mm to 1200 mm. The structure of the in-pipe inspection robot consists of the front and rear driving parts, with the inspection module located centrally. The robot is powered by 22 motors, including eight wheels with motors positioned at both the bottom and the top for propulsion. To ensure that the robot’s center aligns with that of the pipeline during operation, lifting units have been incorporated. The robot is equipped with cameras and LiDAR sensors at the front and rear to monitor the internal environment of the pipeline. Pipeline inspection is conducted using the MFL inspection modules, and the robot’s driving mechanism is designed to execute spiral maneuvers while maintaining contact with the pipeline surface during rotation. The in-pipe inspection robot is configured with wireless communication modules and batteries, allowing for wireless operation. Following its development, the inspection robot underwent driving experiments in actual pipelines to validate its performance. The field test bed used for these experiments is approximately 1 km in length. Results from the driving experiments on the field test bed confirmed the robot’s ability to navigate various curvatures and obstacles within the pipeline. It is posited that the use of the developed in-pipe inspection robot can reduce economic costs and enhance the safety of inspectors when examining aging pipes.

## 1. Introduction

The demand for diverse forms of water supply has surged with industrialization and urbanization. Pipelines serve as the crucial conduit for transporting water to locations where it is desired. Water, an indispensable resource for industrial activities and daily life, is considered a fundamental part of the infrastructure within urban environments. Predominantly, water supply pipelines are installed underground, utilizing materials such as steel and cast iron. In response to the escalating water demand due to industrialization and urbanization in the 1960s, Korea initiated the establishment of multipurpose dams and regional water supply infrastructures, including large-scale waterworks [1]. Presently, industrial and regional water supply pipelines operational since the 1960s are buried underground, with their total length exceeding 4000 km. As of 2005, it was reported that over 32% of pipelines older than 30 years were subject to corrosion, impact damage, and aging [2]. Leakage or damage in water supply pipelines can lead to economic losses and social issues, such as water supply disruptions and sinkhole occurrences. Consequently, research is underway to assess the integrity of aging pipelines and determine the optimal timing for their replacement, aiming to prevent incidents caused by their deterioration.

Currently, commonly used non-destructive inspection methods for pipelines include penetrant testing (PT), radiographic testing (RT), and ultrasonic testing (UT) [3,4,5,6,7,8]. Penetrant testing involves spraying fluorescent material on the surface of the pipeline under inspection and using ultraviolet light to detect defects. This method requires inspecting both the interior and exterior of the pipeline separately. 

Radiographic testing using X-rays or other radiation for flaw detection is expensive and entails radiation exposure risks, rendering it unsuitable for examining water pipes, which are typically constructed with welded sections spanning approximately 9 m. Ultrasonic testing requires close contact with the surface of the object under inspection and is affected by the distance between the pipeline and the inspection equipment. Moreover, it is not feasible for inspecting pipelines that are dirty or coated. 

Non-destructive inspection methods are primarily employed for the examination of water supply pipelines. Notable techniques include visual inspection, ultrasonic testing, MFL detection, and electromagnetic acoustic transducer (EMAT) inspections [9,10,11,12,13]. Traditional inspections of aging buried pipelines often require heavy machinery to unearth the pipelines or personnel entry into the pipelines, posing significant safety risks and financial costs. As a result, the development of a pipeline inspection robot system, equipped with non-destructive testing devices, is underway to facilitate the examination of aging pipelines without these drawbacks.

The deterioration of pipelines outside the buried environment is predominantly detected using magnetic flux leakage (MFL) [14,15,16,17]. Robot-assisted inspection is emerging as a viable alternative, offering operational cost savings and reducing the risk of human casualties. Significant research has been dedicated to the development of pipe inspection robots. In 2011, Kim introduced an optimal mechanism with a propulsion system capable of navigating through steel pipes ranging from 600 mm to 800 mm in diameter [18]. Mateos (2012) unveiled a robot featuring six-wheeled legs designed for movement along the central axes of pipes [19,20]. Wentas (2020) developed a long-range pipeline exploration robot equipped with three continuous tracks [21].

The inspection robot described in this paper utilizes MFL for inspection. MFL allows for defect detection without being influenced by the external environment of the pipeline and is considered the most suitable method for measuring thickness changes in aging pipelines. The MFL inspection method is predominantly utilized in in-pipe inspections among other non-destructive testing techniques. This MFL inspection method possesses the advantage of rapidly inspecting a wide area compared to other non-destructive testing methods. Additionally, unlike other non-destructive testing methods, it does not require a separate surface treatment process (UT, RT) for the inspection target. Due to these advantages, the MFL inspection method is primarily employed in the inspection of gas pipelines, steel pipes, and similar structures [22,23,24,25,26,27,28].

Research on pipeline inspection systems utilizing the MFL method has been actively conducted due to its advantages. Furthermore, various studies are underway to enhance the reliability of pipeline inspection systems using MFL. Zhang [29] proposes an adaptive channel equalization method using a finite impulse response filter to resolve channel mismatches in MFL inspection. Experimental results confirm its efficacy. Salama [30] examined the quantification of uncertainties in MFL intelligent pigs, predominantly used for pipeline inspection, by conducting a full-scale pull-through test on a 12-inch pipe to assess detection and sizing uncertainties. Pham [31] studied the sensitivity of MFL measurement to pipeline magnetization, which is crucial for pipeline inspection. Pham [32] also proposed an ultrasensitive planar Hall magnetoresistive (PH-MR) sensor in an exchange-biased multilayer structure for highly sensitive MFL pipeline inspection.

In the industrial sector, numerous mature systems have been deployed for inspecting defects in long pipeline systems. These systems integrate full-array MFL sensors to rapidly inspect pipe conditions [33,34,35]. However, for in-pipe inspection robots traversing large pipelines, the excessive contact force from fully arrayed MFL sensors with permanent magnets significantly burdens the robot’s drive system. Therefore, we aimed to address this issue by implementing rotating MFL sensors confined to specific areas. This rotational MFL configuration allows us to mitigate the contact force issue, thereby minimizing strain on the robot’s drive system. Although this rotational MFL configuration may result in slower inspection speeds compared to traditional pigging systems, it enabled us to reduce power consumption and the system weight of the robot. Applying defect detection through MFL-based inspection robots is expected to reduce costs and the manpower required for inspections of buried pipelines.

This paper presents the development of a self-propelled pipeline inspection robot equipped with an MFL sensor module, tailored for inspecting aging water supply pipelines. The robot is designed for use in pipes with diameters ranging from 900 mm to 1200 mm.

## 2. Composition of In-Pipe Inspection Robot

### 2.1. Design Constriaints of an In-Pipe Inspection Robot

In this paper, our objective is to develop an in-pipe inspection robot suitable for large pipes with diameters ranging from 900 mm to 1200 mm. The design of this robot must adhere to specific size and composition requirements dictated by the characteristics of the pipes in which it will operate. In Korea, large-scale pipelines are manufactured according to KS D 3578 standards [36]. These pipelines feature continuous miter bands with a 22.5° angle. To navigate the interior of such pipelines, the in-pipe inspection robot must maneuver within a space narrower than the pipeline diameter, particularly when encountering changes in angles. Considering the length of the in-pipe inspection robot, it was determined that the maximum angle variation of the pipeline is 45°. Therefore, the exterior dimensions of the inspection robot were designed to enable navigation even within a 45° angle change in a 900 mm pipeline. While large pipes with a diameter of 900 mm or greater typically do not exhibit abrupt changes in orientation, the robot must be capable of navigating through pipes with 45° miter bends and maneuvering in pipes shaped like a 22.5° equilateral triangle. The design of the inspection robot system must be executed with these constraints in mind.

### 2.2. Composition of In-Pipe Inspection Robot

**Figure 1:**
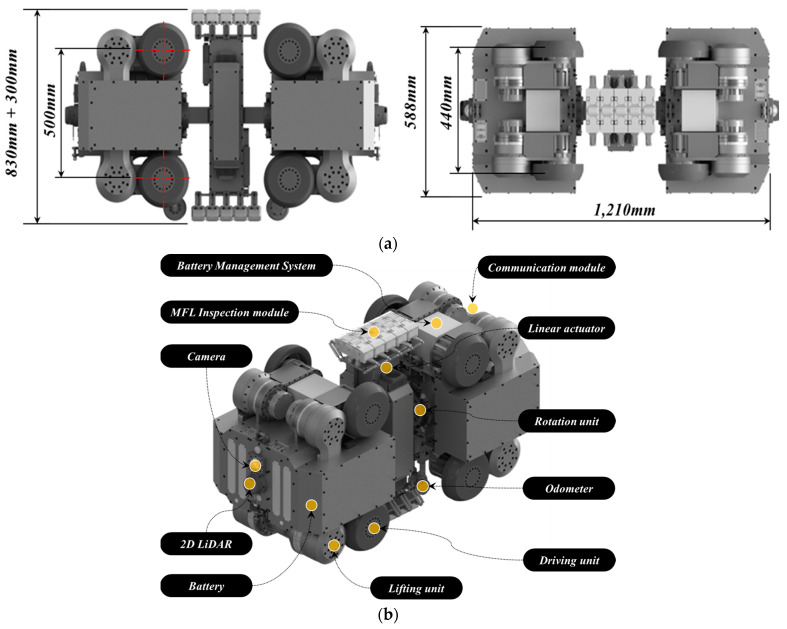
Overview of composition for in-pipe inspection robot. (**a**) Detail dimension of in-pipe inspection robot. (**b**) Configurations of in-pipe inspection robot.

The in-pipe inspection robot system is comprised of two primary components: the driving parts and the inspection part. Figure 1 shows the configuration and dimensions of the in-pipe inspection robot, providing a visual representation of its structure and the spatial arrangement of its components.

- Driving Parts: Each driving segment includes four lifting arms and four driving units. The lifting arms are designed to adjust the robot’s position, ensuring it remains centered within the pipe during the inspection process. The driving units facilitate the robot’s movement through the pipe. To monitor the internal conditions of the pipe and assess the robot’s positioning, each driving part is equipped with a camera module and a 2D LiDAR sensor. The rear driving module features a wireless communication module for external communications. The distance traveled by the robot is measured by odometers attached to the bottom of each driving part. Additionally, an Attitude and Heading Reference System (AHRS) sensor is installed to monitor the yaw, roll, and pitch angle variations of the driving parts.
- Inspection Part: This component is capable of rotating around the pipe’s circumference via a rotation unit, allowing for a thorough circumferential inspection. A linear actuator is integrated to accommodate varying pipe diameters, ensuring compatibility across the specified range. The MFL inspection module, positioned at both ends of the inspection part, includes five inspection units, each equipped with seven Hall sensor modules. This arrangement facilitates comprehensive assessment of the pipe’s condition.

### 2.3. Design of In-Pipe Inspection Robot

The design of the in-pipe inspection robot’s driving system was specifically tailored to accommodate the conditions encountered within large pipes. Considering that these pipes, with diameters of 900 mm or more, do not experience sudden changes in slope and have a maximum slope angle of 22.5°, the driving system was engineered to meet these specific challenges. The required torque for the robot’s movement within the pipe was meticulously calculated, guiding the design of the driving module to ensure efficient navigation through such environments.

- Driving Units: The robot’s driving module features driving units constructed from urethane wheels mounted on an aluminum base, powered by rotation motors. These units are further equipped with harmonic drives and encoders to ensure precise movement and control.
- Lifting Units: Integral to adjusting the robot’s vertical position, the lifting units consist of rotation motors, encoders, harmonic drives, and torque sensors. This setup enables the robot to maintain its central alignment within the pipe, which is crucial to an accurate inspection.
- MFL Rotation and Lifting Units: The design incorporates a hollow structure for the MFL rotation unit, facilitating the passage of communication and power lines, with a slip ring ensuring uninterrupted communication with the driving modules. It includes a rotation motor and a harmonic drive for efficient rotational movement. The MFL lifting unit is designed for vertical extension up to 150 mm, accommodating pipes of various diameters. It comprises a rotation motor, harmonic drive, linear guide, ball screw, and load cell for precise control and force measurement.
- MFL Inspection Modules: To inspect the defects in pipelines, the in-pipe inspection robot has two inspection modules. These modules measure MFL using permanent magnets and Hall sensors. The inspection modules are located at the central ends of the robot. The inspection modules located at each end consist of five independent inspection units. Each inspection unit is equipped with seven Hall sensor modules. One Hall sensor module comprises seven Hall sensors, hence, one inspection module contains a total of 35 Hall sensors. The inspection units applied to the inspection modules come into contact with the pipeline and rotate. During this rotation, the gap distance between the pipeline and the inspection unit affects the inspection quality. To maintain a constant gap distance between the pipeline and the inspection unit, a rotation/suspension mechanism is applied. Furthermore, to reduce the vibration of the MFL inspection module rotating while in contact with the inner surface of the pipeline, individual inspection units are equipped with rotation pivots and suspensions. The pipeline and inspection units rotate with low friction using ball casters. A mechanism capable of linear height adjustment is applied to the sensor part where the Hall sensors are located to maintain a constant distance from the inspected pipeline. Ceramic tips are applied to the end of the sensor module that contacts the pipeline to minimize wear. This design of the inspection module minimizes noise caused by vibration, a drawback of the MFL inspection method performed while in contact with the pipeline, and ensures a constant gap distance between the pipeline and the permanent magnets, resulting in high-quality inspection results.

**Figure 2:**
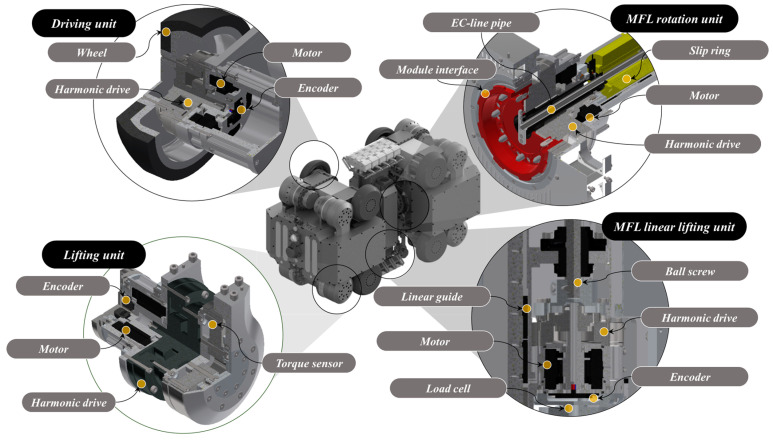
Detailed design of the in-pipe inspection robot.

**Figure 3:**
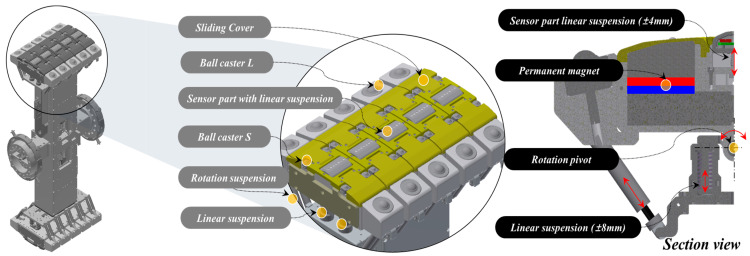
Detailed design of MFL inspection modules.

Figure 2 presents the detailed design of the in-pipe inspection robot, showcasing the integration of these components. Figure 3 shows the detailed design of the MFL inspection modules for the robot.

**Figure 4:**
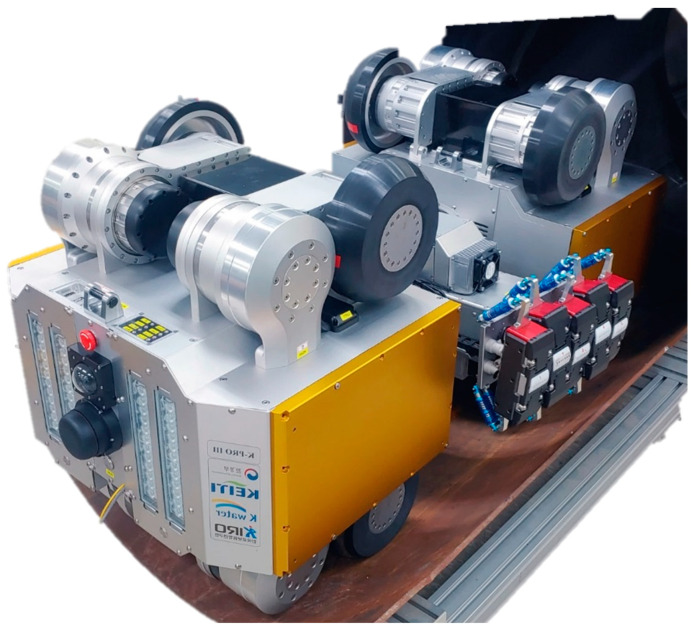
Developed in-pipe inspection robot.

Following the detailed design phase, a prototype of the in-pipe inspection robot was constructed. The final weight of the robot, inclusive of the battery, was recorded at 280 kg, demonstrating the feasibility of the design. Figure 4 illustrates the developed in-pipe inspection robot, highlighting its physical configuration.

**Figure 5:**
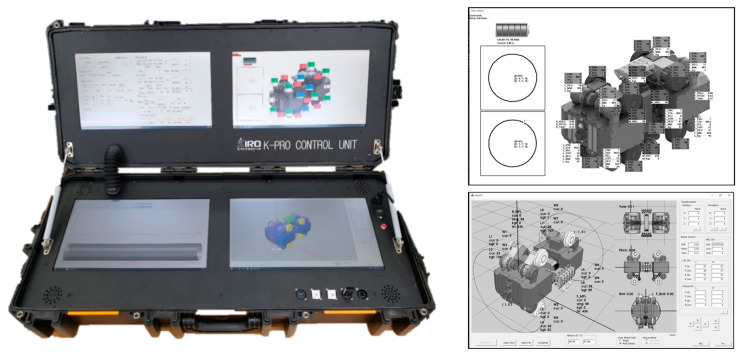
Robot operating system for in-pipe inspection robot.

To facilitate the field test of the developed pipe inspection robot, a Robot Operating System (ROS) was designed. The ROS was designed in a compact form factor to enable easy portability, considering its application in various field environments. Encased within dimensions of 1000 mm (L) × 448 mm (W) × 169 mm (H), the ROS system incorporates four 17-inch touchscreen monitors. Each of these monitors is dedicated to specific functionalities: robot control GUI, robot posture verification monitoring system, robot status monitoring system, and pipe traversal status monitoring system. The ROS system and the robot itself are configured to communicate wirelessly with each other. Figure 5 shows the developed ROS with the GUI environment applied to the ROS depicted on the right side.

## 3. Control of In-Pipe Inspection Robot

The developed in-pipe inspection robot is equipped with a total of 22 motors. The pipeline inspection robot utilizes all 22 motors to drive and inspect the pipeline. In this chapter, we explain how each motor is controlled.

### 3.1. Control of Driving Wheel

The in-pipe inspection robot has four drive wheels on each of its two driving modules. Consequently, the pipeline inspection robot has a total of eight drive wheels for driving. These drive wheels consist of four wheels in contact with the upper part of the pipeline and four wheels in contact with the lower part. The motors of the drive wheels are initially set to a speed control mode to move at a constant speed. However, since the wheels of the inspection robot traveling inside cylindrical pipelines have varying wheel radii depending on the contact area, even if the RPM of each wheel is the same, the actual driving speeds may differ. This results in increased resistance and higher current consumption. Therefore, to minimize the overall power consumption, motors consuming more current than the average current consumption of all wheels have their speeds reduced, while motors consuming less current than the average have their speeds increased.

### 3.2. Control of Odometer Arm

The in-pipe inspection robot is equipped with two odometers, installed under the front and rear driving modules, to measure the robot’s movement distance. During operation, the in-pipe inspection robot controls its posture to align with the pipeline’s center, causing the position of the pipeline bottom and the robot center to fluctuate continually. To accommodate the changing relative height between the pipeline and the robot, the odometers applied to the robot are equipped with mechanisms for height-direction control. These mechanisms are configured to maintain a constant frictional force between the pipeline and the odometer wheel to minimize odometer slippage; thus, they are set to torque control mode. Consequently, regardless of the robot’s height variation, the odometers consistently apply the desired force to the pipeline, enabling a more accurate estimation of the robot’s movement distance.

### 3.3. Control of Driving Lifting Unit for the Driving Wheels

The developed in-pipe inspection robot has 8 lifting units to operate in various pipe environments ranging from 900 mm to 1200 mm in diameter. Through control of the lifting units, the robot ensures that the drive wheels can contact the inner surface of the pipe, from pipes of 900 mm to those of 1200 mm in size. The lifting units applied to the robot consist of 4 units on the upper side and 4 units on the lower side. The upper lifting units are utilized to adjust the vertical resistance of the drive wheels, serving the purpose of increasing vertical resistance when wheel slippage occurs during incline traversal. This is done to enhance friction between the inner surface of the pipe and the wheels, enabling movement. When moving horizontally, minimal force is applied to reduce power consumption of the wheel lift motors. Therefore, the motors of the upper lifting units are set to torque mode, controlling them to exert a constant torque externally as follows.

Where *F_motor_* represents the ultimate input to the motor, while *F_PID_* serves as the control input aimed at achieving the desired torque. Additionally, *F_friction_* denotes the input for compensating for friction, and *F_gravity_* signifies the input for compensating for gravity.

*F_motor_* = *F_PID_* + *F_friction_* + *F_gravity_*

where *F_motor_* represents the ultimate input to the motor, while *F_PID_* serves as the control input aimed at achieving the desired torque. Additionally, *F_friction_* denotes the input for compensating for friction, and *F_gravity_* signifies the input for compensating for gravity.

**Figure 6:**
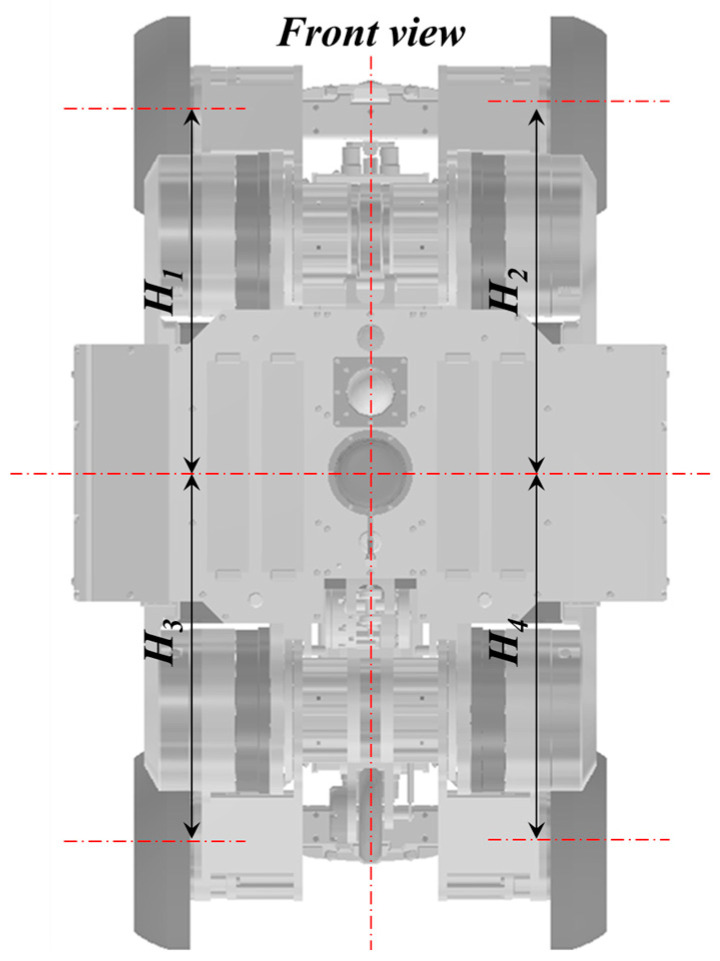
Control parameter description of the lifting unit.

The lifting units located on the lower side are used for maintaining the robot’s horizontal position and aligning the center of the pipe with the robot’s center. Therefore, they employ position control using speed control mode. Since the upper lifting units consistently exert a constant force in the direction of the pipe, the lifting height of the upper lifting units is determined by the lifting height of the lower lifting units. Consequently, utilizing the equation presented below enables precise control of the height for each lower lifting unit, ensuring that the robot’s center maintains alignment with the pipeline’s center throughout its operation. Figure 6 shows the control parameter description of lifting unit control.

*H_d(t_*_−*1)*_ = *(H_1_* + *H_2_)*/*2*

*H_c(t_*_−_*_1)_* = *(H_3_* + *H_4_)*/*2*

*H_c(t)_* = *H_c(t__−__1)_* + *K_p_(H_d(t_*_−_*_1)_* − *H_c(t_*_−_*_1)_)*

Here, *H_d_* represents the reference height of the upper lifting unit for centering, and *H_c_* stands for the current height of the lower lifting unit. By controlling the heights of the upper and lower lifting unit to align them, centering is maintained. *K_p_* denotes the proportional control gain.

### 3.4. Control of MFL Inspection Module

The developed in-pipe inspection robot maintains contact between the MFL inspection module and the inner surface of the pipe while traveling inside. During traversal, the MFL inspection module rotates and moves forward. To achieve this, two drive modules are employed to rotate the MFL module, each equipped with an independent motor, necessitating a control algorithm to synchronize the two modules. The primary objective of this control algorithm is to maintain the angular velocities of the two motors for the MFL rotation module when subjected to external forces. Due to the reactive forces of the two different MFL rotation modules, the roll angles of the two modules can change. The following algorithm enables the MFL rotation module to maintain a specific speed.

**Figure 7:**
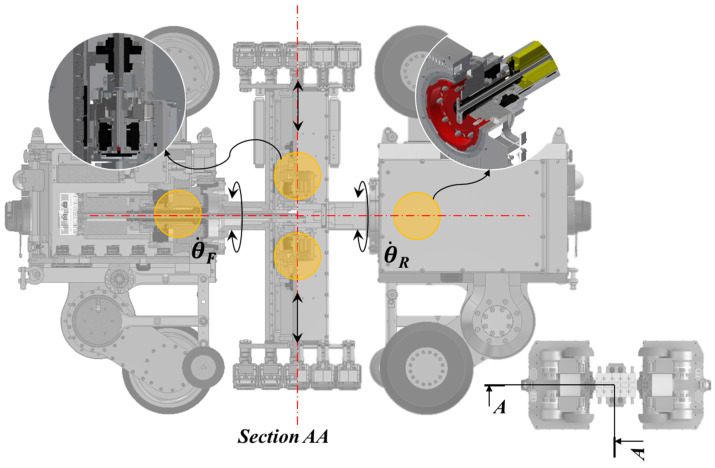
Control parameter description of the MFL inspection module.

The concept of this algorithm is that the rear module assists the front module while the MFL rotation motor mounted on the front driving module rotates the MFL inspection module at a specific speed. Both modules are set to speed control mode, and the objective of this algorithm can be achieved using current feedback. The following equation illustrates the algorithm used here. Figure 7 shows the compositions of the MFL inspection module.

$$\begin{array}{c} {\dot{\theta }}_{F}={\dot{\theta }}_{d}\\ {\dot{\theta }}_{R}={\dot{\theta }}_{d}-K(I_{F}-I_{R}) \end{array}$$

In this equation, ${\dot{\theta }}_{F}$ and ${\dot{\theta }}_{R}$ represent the angular velocities of the front and rear driving modules, respectively, while ${\dot{\theta }}_{d}$ represents the desired angular velocity of the MFL module. *K* is the proportional control gain, and *I_F_* and *I_R_* are the time constants of the front and rear driving modules, respectively. Applying this algorithm resolves synchronization and external force issues for the two MFL rotation modules.

**Figure 8:**
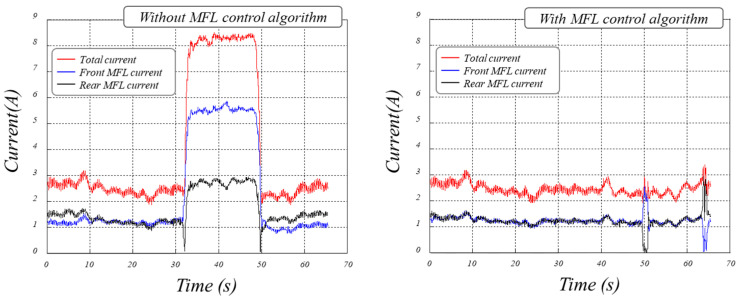
Results of control of rotation direction for MFL inspection module.

To validate the proposed MFL rotation module control algorithm, external forces were applied to the wheel drive units while operating the MFL rotation module. Without the proposed algorithm, it was observed that an increase in current occurs due to the angular difference between the two drive motors applied to the MFL rotation module. However, when the proposed algorithm is applied, it can be noted that the speed of the MFL rotation module adapts immediately to external forces, minimizing the current while maintaining the desired speed. Figure 8 shows the test results of the proposed algorithm for controlling the MFL rotation module.

### 3.5. Control of MFL Inspection Module Lifts

The developed in-pipe inspection robot operates by rotating the MFL inspection module through two MFL rotation units. When the robot travels along the pipeline, there are instances where the robot’s center does not align with the center of the pipeline. Therefore, a controller was designed to comply with disturbances by generating a certain amount of force in the direction indicated when the observed load values measured by the load cell installed on the MFL rotation unit exceed a specific threshold.

**Figure 9:**
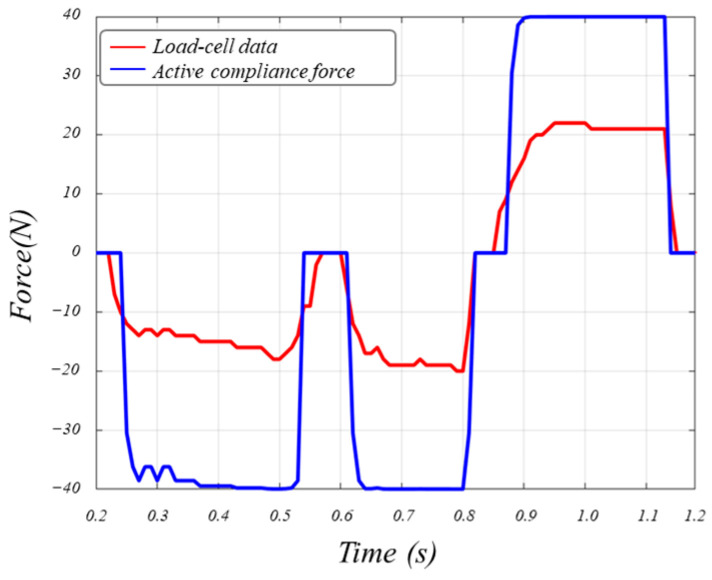
Results of control of linear direction for MFL inspection module.

Figure 9 shows the output of the designed controller for arbitrary disturbances. By using such a controller, it is possible to reduce the load on the motor rotating the MFL by setting the pulling force that reduces the force of the magnets adhering to the pipeline.

### 3.6. Posture Control of In-Pipe Inspection Robot

To operate the in-pipe inspection robot effectively, it must autonomously navigate along predefined paths. During traversal, the robot must maintain a horizontal orientation relative to the pipe to facilitate inspection using MFL. Therefore, horizontal control of the inspection robot is crucial for conducting inspections while traversing. The robot’s orientation is determined through feedback from an IMU (for roll) and LiDARs (for pitch and yaw), and the height information of each wheel lift is utilized to ascertain its horizontal alignment. The attitude accuracy of the IMU is ±0.25° RMS.

#### 3.6.1. Pitch Control of the In-Pipe Inspection Robot

**Figure 10:**
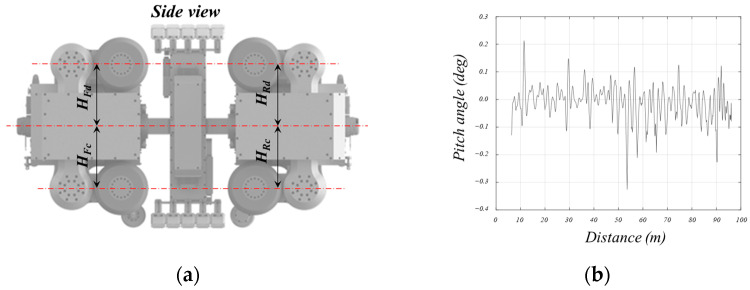
Control parameter description and result of the pitch direction for robot posture.

The purpose of pitch control is to align the heights of the upper and lower lifting units positioned at the front and rear, as depicted in Figure 10a. This control aims to center the front and rear modules individually, ultimately ensuring that the robot aligns with the pipe horizontally.

Figure 10b shows the pitch variation of the robot when moving approximately 100 m inside the pipe using the method. The absolute pitch angle of the robot was measured by the IMU inside of the robot. As observed in the figure, the pitch variation occurring during the robot’s traversal was maintained to within 0.1 degrees. Here, ***H_Fd_*** represents the height of the upper lifting unit at the front, ***H_Fc_*** represents the height of the lower lifting unit at the front, ***H_Rd_*** represents the height of the upper lifting unit at the rear, and ***H_Rc_*** represents the height of the lower lifting unit at the rear.

#### 3.6.2. Roll Control of the In-Pipe Inspection Robot

The developed in-pipe inspection robot contacts the pipeline using the MFL and performs inspections while rotating it. To determine the robot’s orientation, a reference angle is required, typically using the direction opposite to gravity as the reference. The robot’s roll is detected through an IMU, and if this roll does not align with the horizontal, the exact position of defects cannot be accurately determined. Therefore, by adjusting the height (*H_c_*) of the lower lifting unit for centering, the robot’s roll can be controlled using the following equation.

*H_3_* = *H_C_* + *H_PID_ (roll)*

*H_4_* = *H_C_* − *H_PID_ (roll)*

Here, *H_PID_* represents the output of the PID control to make the roll angle zero. *H_1_*, *H_2_*, *H_3_*, *H_4_* are the heights of each wheel lift module when viewed from the front of the robot, as shown in the figure. This control algorithm is applied to both the front and rear drive modules, similar to pitch control.

**Figure 11:**
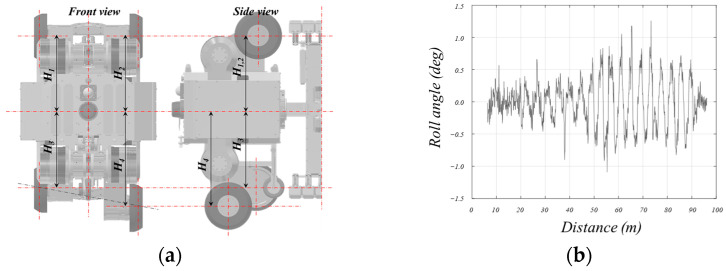
Control parameter description and result of the roll direction for robot posture.

Figure 11 shows the variation in roll angle when moving approximately 100 m inside the pipe using the method. The absolute roll angle of the robot was measured by the IMU inside of the robot. As shown in the figure, it can be observed that the roll angle was maintained to within 0.5 degrees.

#### 3.6.3. Yaw Control of the In-Pipe Inspection Robot

**Figure 12:**
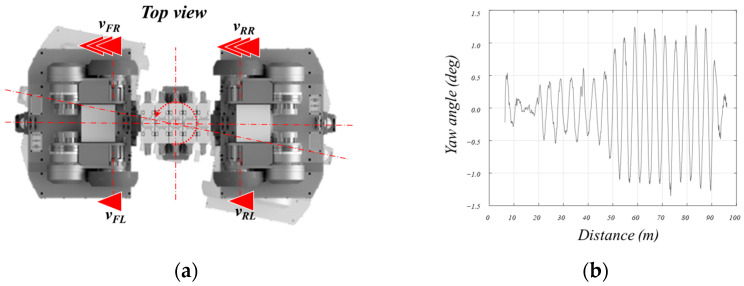
Control parameter description and result of the yaw direction for robot posture.

Yaw control is a crucial element for the robot to move forward along the pipeline’s interior. Initially, the distances on the left and right sides of the robot are measured using the lidar in the front. Yaw control should minimize this distance difference. The yaw control is implemented using the skid-steering method, which utilizes the speed difference between the two wheels as shown in Figure 12a to control the orientation of the robot.

At this point, the angle of yaw requiring correction is measured using LiDARs attached to the front and rear of the robot with respect to the pipeline. By designating the speeds of each wheel according to the following equation, yaw is constantly controlled to be parallel to the axis of the pipeline.

*v_out_* = *K_P_(*−*yaw)*

*v_FL_* = *v_RL_* = *v_desired_* + *v_out_*

*v_FR_* = *v_rr_* = *v_desired_*− *v_out_*

Here, *v_out_* represents the speed output for yaw control, and *K_P_* is the proportional control gain. Therefore, the speed of each wheel is controlled for yaw by adding the control speed output value to the left wheel and subtracting it from the right wheel relative to the desired speed (*v_desired_*) set by the user.

Figure 12b shows the variation in yaw when moving approximately 100 m inside the pipeline using the yaw control algorithm. The yaw of the robot relative to the pipe axis was measured using both LiDARs of the robot. This was achieved by measuring the distances from the center of the robot to both the left and right sides using both the front and rear LiDARs. This method allows for a simple measurement of the robot’s yaw. As depicted in the figure, the yaw control was observed to be accurate to within 1.0 degree.

## 4. Field Testing of In-Pipe Inspection Robot

### 4.1. Configuration of the Field Test Bed

**Figure 13:**
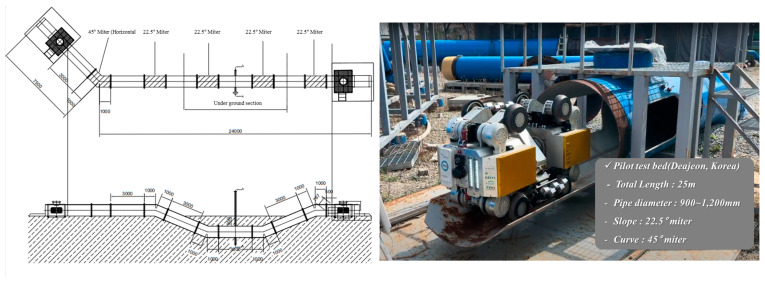
Layout of the pilot test bed for in-pipe inspection robot.

Before the field test of the in-pipe inspection robot we developed, a pilot test was performed in the laboratory. The pilot test bed was designed to simulate conditions similar to those of large-diameter buried pipes. The pilot test bed comprised sections including a horizontal 45-degree mitered pipe and a sloped section with a 22.5-degree incline. The pipes constituting the pilot test bed were sized with diameters ranging from 900 mm to 1200 mm. Through the pilot test bed tests, it was ensured that the developed pipe inspection robot would be operable in real-world environments. According to the pilot test bed tests, it was confirmed that the design maximum speed of the in-pipe inspection robot, set at 300 mm/s, was achievable. Based on driving tests on the pilot test bed, it is inferred that the developed in-pipe inspection robot will be capable of operating in actual pipelines with various forms of curvature. Figure 13 shows the configurations of the pilot test bed located at Deajeon, Korea.

**Figure 14:**
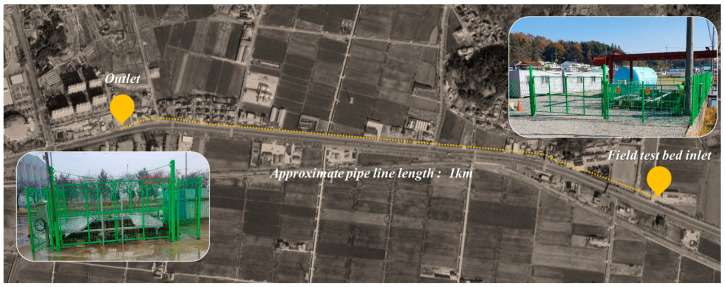
Overview of the field test bed for in-pipe inspection robot.

The field test of the in-pipe inspection robot was conducted at a field test bed located in Gunsan, Korea. The pipeline spanned approximately 1 km, featuring diverse configurations of curves and gradients. The field test bed for the in-pipe inspection robot driving test is buried at a depth of 6 m underground. With detailed information lacking about the buried pipeline, the aim was to acquire detailed information as part of the robot’s driving test. Figure 14 shows the overview of the field test bed location and layout.

**Figure 15:**
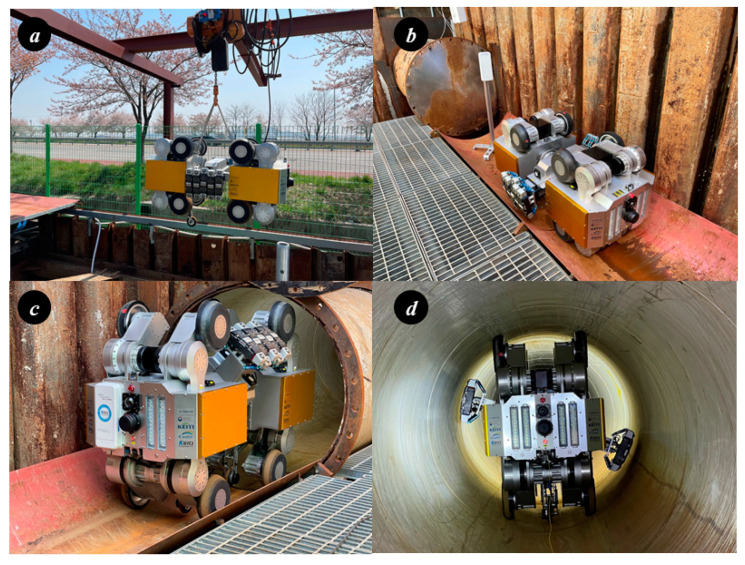
Driving test of in-pipe inspection robot at field test bed (located at Gunsan).

Figure 15 shows the field test of the pipe inspection robot conducted on the test bed. Figure 15a shows the movement of the robot through an inlet located underground using a winch. Figure 15b shows the setup of the robot on a cut section of the pipe for inspection purposes. Figure 15c shows the robot entering the pipe to begin the inspection. Figure 15d shows the robot performing posture control after entering the pipe and conducting the driving test. The driving test of the in-pipe inspection robot at the field test bed was conducted without battery replacement and recharging. Due to the absence of detailed information regarding the specific location and configuration of the target pipeline, the signals obtained during the robot driving test allowed for the assessment of the shape and composition of the pipeline.

### 4.2. Results of Driving Test of In-Pipe Inspection Robot at Field Test Bed

**Figure 16:**
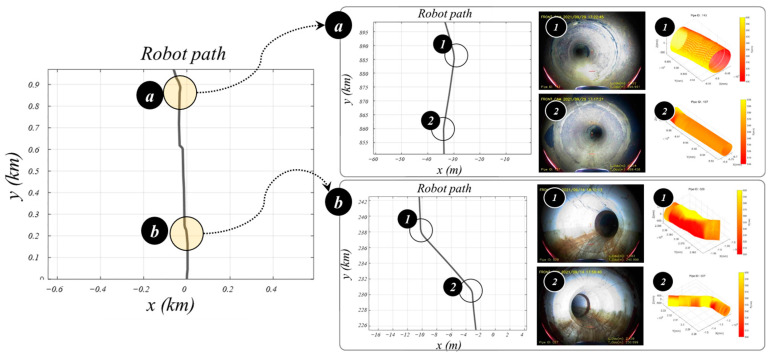
Robot path and pipeline environment in driving test on the field test bed.

Throughout the driving tests conducted on the field test bed, the LiDAR located at the front and rear center of the robot was utilized to assess the condition of the pipe, detect obstacles, and determine the relative positions of the robot and the pipe. By employing LiDAR on the robot, the distance between the robot and the center of the pipe was calculated, and the lifting arm and driving wheels were controlled to ensure that the robot remained positioned at the center of the pipe during navigation. The point cloud data obtained through LiDAR can be transformed into a three-dimensional graphic with the pipe center as the reference coordinate system, allowing visualization of the pipe’s internal condition. Figure 16 shows the path followed by the robot while navigating the entire length of the actual pipe on the field test bed. The total length of the pipe was approximately 980 m, calculated using the odometer mounted on the robot’s underside. The curvature inside the pipe was determined using a combination of signals from the IMU sensor and the odometer mounted on the robot. Figure 16a,b depict the shapes of the curves present in the pipe on the field test bed. The condition of the pipe can be observed using the camera mounted on the robot, as shown in Figure 16(a1,b1), while the overall shape of the pipe can be determined using LiDAR, as shown in Figure 16(a2,b2).

**Figure 17:**
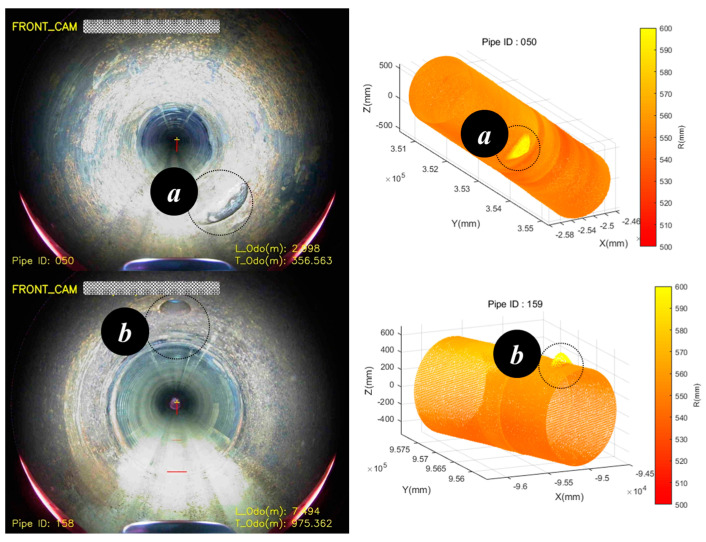
Configuration of branch pipes (obstacles) confirmed using cameras and LiDAR.

In large-scale pipelines utilized as water supply infrastructure, various forms of branch pipes are often present within the pipeline network. When conducting inspections using in-pipe inspection robots, it is imperative to anticipate and address obstacles such as branch pipes in advance. Thus, this study sought to ascertain the feasibility of detecting obstacles, including branch pipes, through the utilization of LiDAR sensors integrated into the inspection robots and to validate their ability to navigate past detected obstacles. Upon encountering branch pipes within the pipeline, the robot distinguishes between the wheels in contact with the pipe’s interior and those not in contact, subsequently maneuvering through controlled motions to surmount these obstacles. Figure 17 shows the shapes of branch pipes detected during field test bed experiments. The detection of branch pipes via LiDAR mounted on the in-pipe inspection robot has been confirmed. The developed in-pipe inspection robot has been confirmed to be capable of obstacle traversal through experiments.

The field test bed operation lasted a total of 4 h. The average driving speed of the pipeline inspection robot was determined to be 69.4 mm/s. During a long-distance drive of 1 km, the pipeline inspection robot encountered no communication issues, and it was confirmed that inspections of pipelines exceeding 1 km in length can be conducted without the need for battery replacement.

Typically, meticulous examinations are reserved for pipelines aged beyond 30 years, with subsequent determinations of the need for partial repair or complete replacement made upon defect identification. The decision to replace the pipeline is made when a thickness reduction of more than 30% is detected. During the field test, inspections for pipeline defects were conducted using the MFL inspection module. The field test bed consisted of a pipeline with a diameter of 1000 mm and a thickness of 10 mm. To delineate regions necessitating repair or replacement during inspection, assessments were carried out utilizing the inspection module. Throughout these assessments, segments manifesting a decrease in thickness of 20% or more were discerned, factoring in safety margins. The field test bed inspection revealed that a total of 10 segments exhibited a decrease in pipeline thickness of 20% or more. Four segments were identified for repair or replacement due to a thickness reduction exceeding 30% along them. Table 1 provides a comprehensive depiction of the inspection findings from the field test bed.

In Table 1, the position represents the movement distance from the starting position of the robot. The angle is defined as 0° with respect to the vertical direction and 360° when rotated. The Pipe Number is defined as 1 for the pipeline at the starting position of the robot, and subsequent numbers are assigned as the robot moves and detects welding lines.

**Figure 18:**
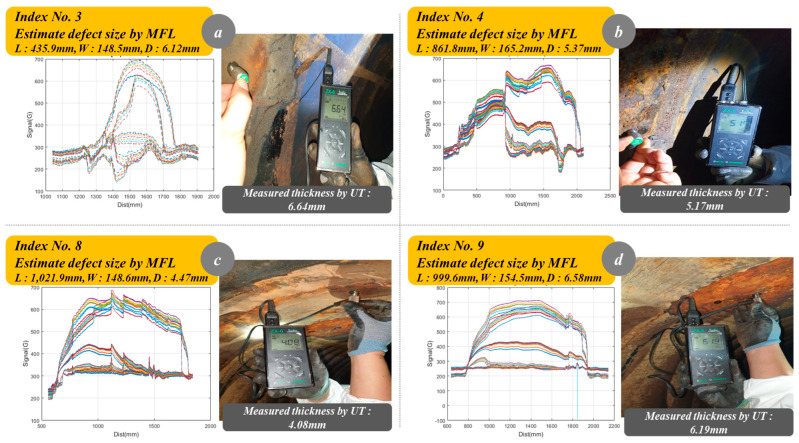
The defect inspection results of the in-pipe inspection robot (field test bed).

Figure 18 is the results of pipeline defects detected through the in-pipe inspection robot. Based on the defect inspection results from the field test bed, repairs and replacements were deemed necessary at 4 locations. Raw data obtained through MFL inspection undergoes noise removal through de-trending and low-pass filtering during the pre-processing stage and is further processed during the post-processing stage to calculate the size of defects and the thickness of the pipeline. In this inspection, areas where the pipeline thickness had decreased by 20% or more were designated as defects. Figure 18a–d depicts the signals of defects detected through the in-pipe inspection robot. The graphs in Figure 18a–d show the signals of 35 Hall sensors positioned in the inspection module. The inspection module can inspect a 250 mm section of the pipeline longitudinal direction with each rotation. During pipeline inspection, the robot travels forward while rotating the inspection module in contact with the pipeline. This robot motion enables the acquisition of spiral-shaped inspection sections. The 35 Hall sensors embedded in the inspection module are spaced at 7 mm intervals. When the inspection module inspects a section with defects, changes in the magnetic flux density can be measured via the Hall sensors. As observed in Figure 18a–d, variations in gauss values measured by the Hall sensors in the inspection module enable the prediction of defect shapes.

Among the 10 detected defects, the 4 sections requiring repair and replacement were re-measured using ultrasonic test equipment (Dakota, CA, USA, ZX-6). The inspection results from the ultrasonic test equipment confirmed a maximum error of 8.72% compared to the MFL inspection results.

Table 2 compares the defect results measured by the in-pipe inspection robot with those measured by the ultrasonic test equipment. The results obtained by the ultrasonic test equipment only measure the thickness of the pipeline. Therefore, the results related to thickness were compared between the two methods of defect measurement. It was confirmed that there is an average error range of 6.72% between the defect measurement results obtained by the in-pipe inspection robot and those obtained through the ultrasonic test equipment.

## 5. Discussion

This study describes the development of a robot system for inspecting water pipelines buried underground. Through testing on a pilot test bed, it was confirmed that the developed pipeline inspection robot can operate within pipelines ranging from 900 mm to 1200 mm in diameter. Furthermore, it was verified that the robot can navigate through pipes with horizontal angles of up to 45° and gradients of 22.5°. A field test bed representing actual water pipeline usage was selected for conducting a 1 km long-distance driving experiment using the developed pipeline inspection robot. However, when employing the developed robot for real-world pipeline driving and inspection, several additional research considerations arise. It is necessary to ascertain whether the robot can overcome obstacles present within the pipeline during navigation. While the mechanism allows the robot to overcome vertical obstacles of less than 50 mm in height during driving, inspection is rendered impossible in sections where obstacles are present. Thus, identification of obstacle location and size is crucial, and inspections should be conducted excluding sections with obstacles. Additionally, to inspect various types of defects occurring in pipelines, standard defects need to be fabricated for comparison with actual pipeline defects. For future improvements in research outcomes, long-distance driving experiments (over 1 km), inspections, and wireless communication experiments on actual pipelines are necessary to ensure the reliability of pipeline inspection robots operating within pipelines for extended periods.

## 6. Conclusions

In this study, we developed a robot system capable of inspecting the interior of large-diameter water pipelines. The designed in-pipe inspection robot consists of three parts. The driving parts, located at the front and rear of the robot, are responsible for propulsion, while the inspection part is positioned at the center for conducting inspections. Within the inspection part, two MFL inspection modules are implemented. Each MFL inspection module comprises five inspection units, with each unit equipped with seven Hall sensors. With the Hall sensors applied, the MFL inspection module can inspect a length of 600 mm along the pipeline with one rotation. A total of 22 motors are employed to drive the robot, with eight motors dedicated to propelling the robot’s wheels and another eight utilized in the lifting units to accommodate various diameters of pipelines (ranging from 900 mm to 1200 mm). The robot is equipped with two cameras and two LiDAR sensors to monitor the internal environment of the pipeline during its operation. The developed in-pipe inspection robot underwent driving experiments on a test bed with diameters ranging from 900 mm to 1200 mm. The results confirmed the robot’s capability to navigate through test beds with various diameters and slope sections. Following completion of control performance experiments, driving experiments were conducted on a field test bed utilizing actual pipelines. The field test bed, measuring a total length of 1 km, enabled the in-pipe inspection robot to traverse the pipeline for 1 km within a duration of 4 h without the need for battery replacement or recharging. It is anticipated that the developed in-pipe inspection robot will enable inspection of aging pipelines and detection of pipeline damage in the future.

## Figures and Tables

**Table 1:** The information of defects detected by the in-pipe inspection robot on the field test bed.

Index	Pipe No.	Position (m)	Angle (°)	Thickness (mm)
1	32	256.54	260.9	7.92
2	37	278.46	252.6	7.97
3 *	52	346.28	285.5	5.17
4 *	52	365.91	270.2	6.64
5	79	525.37	276.6	7.03
6	80	529.11	299.5	7.97
7	105	674.18	274.9	7.86
8 *	149	925.52	296.8	4.08
9 *	155	978.17	329.8	6.19
10	155	980.28	322.3	7.29

* Defect detected locations of thickness reduction over 30%.

**Table 2:** Results comparison between MFL inspection and ultrasonic testing.

Index	Estimated Defect Value (MFL Inspection)			Measured Thickness by Ultrasonic (mm)	Error (%)
	L (mm)	W (mm)	T (mm)		
3	435.9	148.5	6.12	6.64	8.49
4	861.8	165.2	5.37	5.17	3.72
8	1021.9	148.6	4.47	4.08	8.91
9	999.6	154.5	6.58	6.19	5.92

## Data Availability

The original contributions presented in the study are included in the article, further inquiries can be directed to the corresponding author.
